# Clinical characteristics and primary outcomes of patients with ANCA-associated vasculitis and central diabetes insipidus

**DOI:** 10.3389/fendo.2023.1173903

**Published:** 2023-05-12

**Authors:** Xin Chen, Shuo Zhang, Xia Peng, Xiaoxiao Shi, Haiting Wu, Yubing Wen, Yan Qin, Xinping Tian, Huijuan Zhu, Limeng Chen

**Affiliations:** ^1^ Department of Nephrology, State Key Laboratory of Complex Severe and Rare Diseases, Peking Union Medical College Hospital, Chinese Academy of Medical Science and Peking Union Medical College, Beijing, China; ^2^ Department of Rheumatology, Peking Union Medical College Hospital, Chinese Academy of Medical Science and Peking Union Medical College, Beijing, China; ^3^ Department of Endocrinology, Peking Union Medical College Hospital, Chinese Academy of Medical Science and Peking Union Medical College, Beijing, China

**Keywords:** antineutrophil cytoplasmic autoantibody-associated vasculitis, diabetes insipidus, recurrence rate, PR3-ANCA, MPO-ANCA

## Abstract

**Introduction:**

Anti-neutrophil cytoplasmic antibody (ANCA)-associated vasculitis (AAV) is characterized by systemic small-vessel vasculitis and may rarely present as central diabetes insipidus (CDI). In this study, we aimed to determine the clinical characteristics and prognosis of patients with AAV-associated CDI.

**Methods:**

This was a nested case-control study where AAV patients with CDI at the Peking Union Medical College Hospital were followed from January 2012 to April 2022. Case-control matching with AAV patients without CDI was performed (1:5), and participants were matched by age, sex, and AAV classification. We collected clinical data every 3–6 months and conducted a literature review using PubMed to identify relevant articles published from 1983–2022.

**Results:**

Among 1203 hospitalized AAV patients, 16 patients with CDI were included (1.3%). The average age was 49 years, and men accounted for 56.3%. Granulomatosis with polyangiitis (GPA) accounted for 87.5% of patients. AAV patients with CDI had more ear, nose, and throat (ENT) (81.3%) involvement and less renal impairment than those in the control group (P<0.05). After a mean follow-up of four years, 50% of patients were in remission from AAV, 37.5% relapsed, and 12.5% died. Our literature review suggested that patients in Asian countries tend to be older men and have higher myeloperoxidase (MPO-ANCA) positivity than those in Western countries. Furthermore, proteinase 3 (PR3-ANCA) positivity may predict disease recurrence.

**Discussion:**

AAV patients with CDI had more ENT involvement and a higher eGFR. MPO-ANCA positivity is more commonly observed in Asian countries than Western countries, and PR3-ANCA positivity may predict recurrence.

## Introduction

Anti-neutrophil cytoplasmic autoantibody (ANCA)-associated vasculitis (AAV) is a group of disorders characterized by severe systemic small-vessel vasculitis, marked by alveolar hemorrhage and rapidly progressive pulmonary glomerulonephritis (1). AAV includes three subgroups, namely microscopic polyangiitis (MPA), granulomatosis with polyangiitis (GPA), and eosinophilic granulomatosis with polyangiitis (EGPA).

Central diabetes insipidus (CDI) is a rare condition associated with AAV. CDI may precede or follow the diagnosis of AAV (2, 3). The incidence rate of CDI in AAV patients is reported to be approximately 1.3–3.9% by case series and retrospective studies (3–5). The occurrence of DI in patients with AAV might be explained by vasculitis affecting the pituitary vasculature, involvement of the adjacent pituitary gland by granulomatous masses originating from the ear, nose and throat (ENT); and primary granulomatous inflammation (6). Symptoms of CDI can vary from multiple cranial nerve palsies (7) to anterior pituitary dysfunction (8). AAV-associated CDI should be recognized to avoid unnecessary biopsies of the pituitary gland and minimize the risk of irreversible loss of pituitary function.

Previous case reports and series on AAV-associated CDI have no prolonged follow-up and have not delineated the risk factors to predict prognosis. Furthermore, population differences between Asian and Western countries have not been fully elucidated. In this retrospective nested case-control study, we investigated the association between CDI and AAV, considering the disease burden, disease control situation, long-term follow-up, and prognosis. In addition, we analyzed the genetic and clinical characteristics of these patients in China and Western countries by integrating clinical data and conducting a comparative analysis.

## Materials and methods

This was a nested case-control study where AAV patients with CDI at Peking Union Medical College Hospital were followed every 3–6 months from January 2012 to April 2022. This group was matched to a control group of AAV patients without CDI according to a 1:5 ratio. AAV relapse was defined as new evidence of systemic activity, increased inflammatory markers, or re-increase in ANCA titers. Survival analysis was used to investigate the risk factors of disease recurrence in AAV patients with CDI.

### Patients

We enrolled hospitalized AAV patients with CDI at Peking Union Medical College Hospital and followed them from January 2012 to April 2022. The inclusion criteria included a diagnosis of AAV according to the definition by the 2012 revised International Chapel Hill Consensus Conference (9) and a diagnosis of CDI confirmed by the fluid deprivation-vasopressin test. Cases of CDI associated with causes other than AAV, such as tumors or infection, were excluded. Case-control matching with AAV patients without CDI was performed (1:5). Patients were matched according to age and sex by Model 1 and age, sex and AAV classification by Model 2. The study was a retrospective study, and the ethics committee agreed to waive the informed consent. All procedures and laboratory tests were performed according to the national ethical guidelines and approved by the Standing Committee for Clinical Studies at Peking Union Medical College Hospital (No. JS 3527)

### Definitions

The definitions in the inclusion criteria for AAV patients with CDI were as follows: 1) AAV was defined and classified as granulomatosis with polyangiitis (GPA), microscopic polyangiitis (MPA), or eosinophilic granulomatosis with polyangiitis (EGPA) according to the 2012 revised International Chapel Hill Consensus Conference (9); 2) Polyuria was defined as, assessed by a 24 h urine volume of more than 2.5 L; 3) CDI was defined as polyuria with a positive fluid deprivation test (10).

Hypopituitarism refers to a partial or complete failure of secretion of anterior pituitary hormones and includes the following entities: hypogonadotropic hypogonadism (low levels of serum testosterone or estradiol and low or inappropriately normal luteinizing and follicle-stimulating hormone levels), secondary hypothyroidism (low serum thyroxine level and low or inappropriately normal thyroid stimulating hormone deficiency) and secondary hypocortisolism (low morning (0800 h) cortisol and adrenocorticotropic hormone (ACTH) levels).

### Laboratory analyses and radiological assessments

Baseline clinical characteristics such as age, sex, symptoms, system involvement, AAV classification, laboratory results, and magnetic resonance imaging (MRI) were recorded as part of the data collection. Laboratory tests, including blood and urine tests, were performed on the day of admission. ANCA titers were assessed using an indirect immunofluorescence assay and an antigen-specific enzyme-linked immunosorbent assay (ELISA). The estimated glomerular filtration rate (eGFR) was calculated using the Chronic Kidney Disease Epidemiology Collaboration (CKD-EPI) creatinine equation (11). Pituitary enhanced MRI was performed by radiologists, and any abnormalities were reported. The images were evaluated for posterior hyperintense signals, pituitary microadenomas, space-occupying lesions in the posterior lobe, pituitary stalk thickening, and hypertrophic cranial pachymeningitis.

### Treatment and prognosis

We recorded the type of therapy each participant received, including hormone replacement therapy, induction therapy, and maintenance treatment for vasculitis. Clinical characteristics and laboratory indicators were followed up every 3–6 months, and structural and functional assessments of the pituitary gland were recorded. In terms of prognosis, the state of improvement for patients with AAV-associated CDI was defined as a Birmingham Vasculitis Activity Score (BVAS) ≤ 2 after treatment. Recurrence was defined as new evidence of systemic activity, increased inflammatory markers, or re-increase in ANCA levels. The resolved CDI was defined as not requiring the use of desmopressin acetate tablets (DDAVP), improved CDI as reducing the amount of DDAVP and persistent CDI as reliance on DDAVP.

### Statistical analysis

Statistical analysis was performed using SPSS Statistics for Windows (ver. 22, IBM Corp.). Continuous variables were expressed as means with standard deviations or medians with interquartile ranges, as appropriate. Categorical variables were expressed as proportions. One-way analysis of variance (ANOVA) was used to compare the means of more than two groups. Survival analysis was performed to investigate the risk factors for recurrence in patients with AAV-associated CDI. A two-tailed test was used, and statistical significance was set at P < 0.05.

### Literature review

We conducted a systematic literature review using PubMed throughout November, 2022. We used medical subject headings and free-text search terms for our search, which included a manual search for references to existing reviews in the field. The inclusion criteria were as follows: 1) a diagnosis of GPA according to the 2012 revised International Chapel Hill Consensus Conference; 2) a diagnosis of CDI assessed by the 24 h urine output and confirmed by a positive fluid deprivation-vasopressin test; and 3) cases with well-documented clinical information. Patients with secondary vasculitis or other autoimmune diseases were excluded from this study.

## Results

### Baseline clinical characteristics

**Figure 1:**
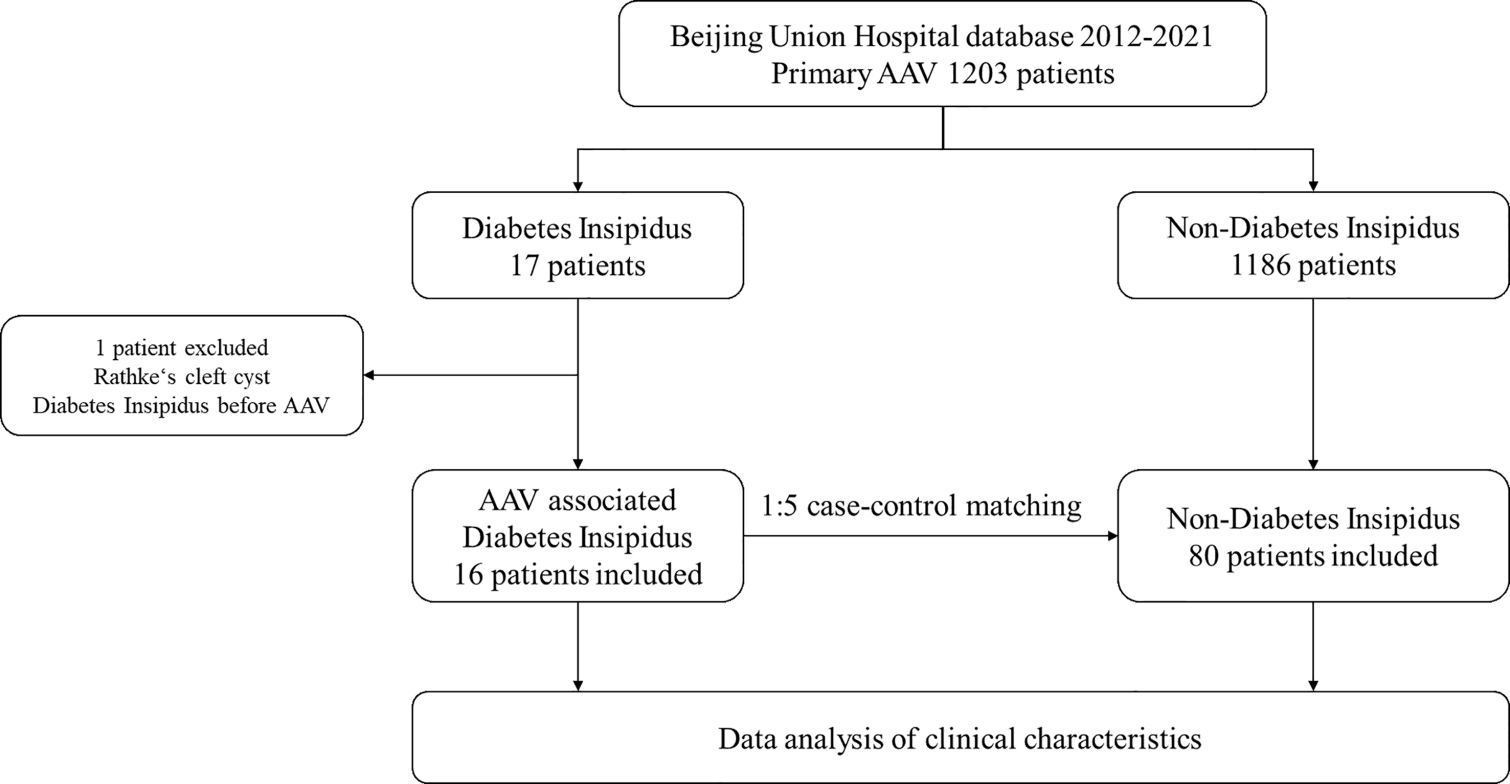
Flow chart. *AAV, ANCA-associated vasculitis.

A total of 1203 patients were included in the study, among which 16 (1.3%) had AAV-associated CDI (**Figure 1**). The average age was 49 years, and men accounted for 56.3%. The average urine output was 7 ± 2 L/24h. GPA accounted for 87.5% of patients, while MPA and EGPA accounted for 6.25% each. Diabetes insipidus was the initial presentation in 50% of patients that sought medical attention. Organ involvement included the ENT (81.3%), eye (62.5%), nervous system (other than CDI) (50%), lung (43.8%), skin (31.3%), and kidney (18.8%) (**Table 1**).

**Table 1:** Baseline clinical characteristics of patients with AAV and diabetes insipidus.

ID	Age	Sex	AAV	ANCA	First symptom	Organ involvement	BVAS
1	49	Female	GPA	MPO	AAV	Lung, ENT, Skin, NS	15
2	62	Female	GPA	MPO	DI	ENT, Eye, NS	15
3	64	Female	GPA	MPO	DI	Lung, ENT	14
4	49	Male	GPA	MPO	DI	ENT, Skin	7
5	50	Male	GPA	MPO	AAV	ENT, Eye	18
6	32	Female	GPA	MPO	DI, Fever	Eye, Skin	10
7	17	Female	GPA	PR3	AAV, DI	Lung, ENT	10
8	54	Male	GPA	PR3	AAV, Fever	Kidney, Lung, Skin, NS, CVS	26
9	54	Male	GPA	PR3	DI	ENT	11
10	46	Male	GPA	PR3	AAV, Fever	Lung, ENT, Eye, Skin	17
11	37	Male	GPA	PR3	AAV, Fever	Kidney, Lung, ENT, Eye	37
12	59	Female	GPA	Negative	AAV, Fever	ENT, Eye, NS	18
13	25	Male	GPA	Negative	AAV	ENT, Eye, NS	16
14	64	Male	GPA	Negative	AAV	Eye, NS	17
15	73	Female	MPA	MPO	DI	Kidney, ENT, NS, GI	12
16	59	Male	EGPA	MPO+PR3	AAV	Lung, ENT, Eye, NS	21

AAV, ANCA-associated vasculitis; GPA, granulomatosis with polyangiitis; MPA, microscopic polyangiitis; EGPA, eosinophilic granulomatosis with polyangiitis; DI, diabetes insipidus; ANCA, anti-neutrophil cytoplasmic antibodies; MPO, myeloperoxidase; PR3, proteinase 3; ENT, Ear, Nose and Throat; NS, nerve system (other than DI); CVS, cardiovascular system; GI, Gastrointestinal; BVAS, Birmingham Vasculitis Activity Score (BVAS) v3.

Endocrinological assessment revealed hypopituitarism in 56.3% of patients, including hypogonadotropic hypogonadism in 25.0% and secondary hypothyroidism in 50.0%. Hypocortisolism, growth hormone deficiency, and hyperprolactinemia were present in 28.6%, 9%, and 44.4% of patients, respectively. MRI abnormalities were observed in 93.7% of patients. Almost all patients lacked posterior hyperintense signals; approximately one-third of them had space-occupying lesions (posterior pituitary occupancy), and one-fifth had pituitary stalk thickening. One patient had done pituitary biopsy with granulomatous inflammation. Follow-up pituitary MRI revealed that the pituitary lesions in seven patients had shrunk after treatment. (**Table S1**–**S3**).

### Treatment and follow-up

All patients were treated with oral prednisone (0.8–1 mg/kg/day), and 50% were administered intravenous methylprednisolone pulse therapy. Immunosuppressants used for inducing remission included cyclophosphamide (CTX, 87.5%), rituximab (RTX, 25%), methotrexate (MTX, 25%), and mycophenolate mofetil (MMF, 12.5%). Maintenance treatment included oral prednisone (81.3%), CTX (43.8%), MTX (25%), MMF (18.75%), azathioprine (AZA) (18.75%), and RTX (6.25%). Over the following 4.14 ± 2.63 years, eight patients showed improvement (50%), six suffered from recurrence (37.5%), and two died (12.5%) from chronic heart failure and infection (**Table 2**).

**Table 2:** Treatment and outcome of patients with AAV and diabetes insipidus.

ID	Treatment			Follow-up	
	Remission induction	Maintenance treatment	DDAVP (mg per day)	Time (yrs)	Outcome#
1	PDN po., CTX, MTX, IVIG, intrathecal IT	No	0.1	0	Dead
2	PDN po., CTX	PDN po., MMF.	0.15	2	Improved
3	PDN po., CTX	PDN po., MMF.	0.15	5	Improved
4	PDN po., CTX	PDN po., CTX, MTX.	0.2	3	Improved
5	PDN po., MMF	MMF	0.075	3	Improved
6	PDN po., CTX, MTX	PDN po., CTX, MTX.	0.3	4	Improved
7	PDN po., MP pulse., RTX, MTX	PDN po.	0.15	1	Improved
8	PDN po., MP pulse., CTX	PDN po., AZA.	0^*^	2	Recurrence
9	PDN po., CTX	PDN po., CTX.	0.25	3	Recurrence
10	PDN po., MP pulse., CTX, RTX, FK506, MMF	PDN po., CTX, MTX.	0.1	10	Recurrence
11	PDN po., MP pulse., CTX, RTX	PDN po., AZA.	0^*^	6	Recurrence
12	PDN po., MP pulse., CTX, intrathecal IT	PDN po., CTX.	0.15	4	Dead
13	PDN po., MP pulse., CTX	PDN po., FK506.	0.15	8	Improved
14	PDN po., MP pulse., CTX, intrathecal IT	PDN po., CTX, AZA.	0.1	4	Recurrence
15	PDN po., CTX	CTX	0.05	6	Improved
16	PDN po., MP pulse., CTX, RTX, MTX, IVIG, intrathecal IT	PDN po., RTX, MTX.	0.1	1	Recurrence

AAV, ANCA-associated vasculitis; DDAVP, desmopressin acetate tablets; MP pulse., intravenous methylprednisolone pulse therapy; PDN po., oral prednisone; RTX, rituximab; CTX, cyclophosphamide; MTX, methotrexate; FK506, tacrolimus/rapamycin; MMF, mycophenolate mofetil; intrathecal IT, intrathecal injection (dexamethasone ± methotrexate); IVIG, intravenous immune globulin; AZA, azathioprine.

^#^Outcome: Improved, BVAS ≤ 2 after treatment; Recurrence, newly diagnosed active symptom of AAV.

^*^Two patients had renal dysfunction (with chronic kidney disease III-IV) and did not take desmopressin with the urine output of 1.5-3L.

**Figure 2:**
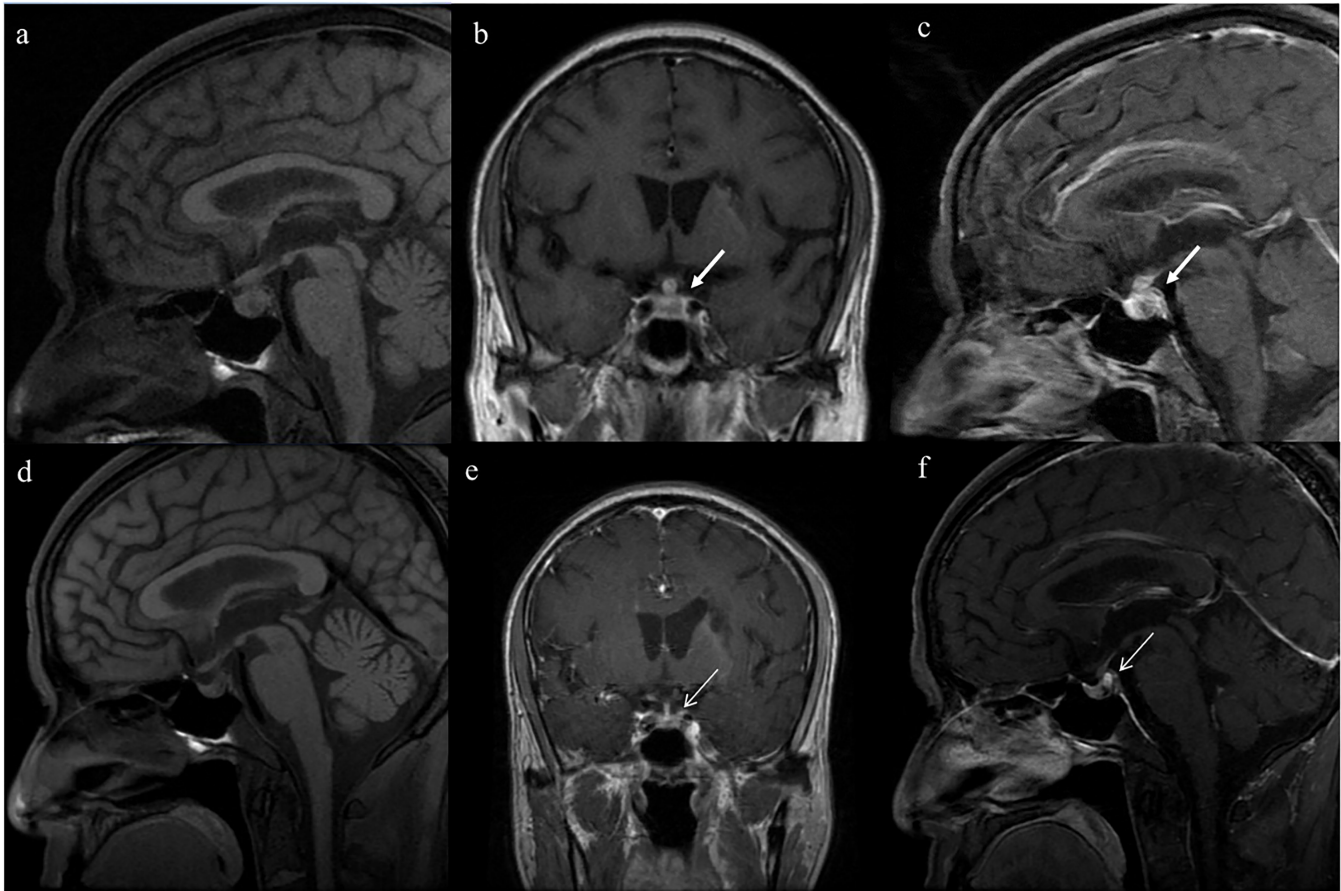
Serial magnetic resonance imaging (MRI) of the pituitary in patient No.9, who fully recovered from diabetes insipidus. Enlargement of the posterior pituitary lobe (5.7mm×7.8mm) and thickening pituitary stalk (transverse diameter 5.3mm) on T1-weighted images (Panels **A–C**; thick white arrow). After the treatment, the enlargement of the posterior pituitary shrank(3.8mm×4.5mm) and the normalization of the pituitary stalk (transverse diameter 2.3mm) (Panels **D–F** thin white arrow).

Almost all patients were administered desmopressin acetate tablets (87.5%), except for those with chronic renal disease who are at risk of substantially decreased urine output with desmopressin. Among the patients treated with desmopressin, 70% showed improvement in polyuria and reduced reliance on hormonal replacement therapy. Imaging follow up was performed in seven out of the sixteen patients. Patient 9 demonstrated complete recovery with improvement in imaging and no longer required desmopressin (**Figure 2**). Imaging revealed improvement with shrinkage of the lesions in two patients. The time span between the start of AAV treatment and improvement of CDI was 28 ± 15 days. Approximately one-fifth of patients with hypogonadotropic hypogonadism showed improvement without the need for hormonal replacement therapy.

### Comparison of patients with GPA-associated CDI between our cohort and the literature review

Among the 14 patients with GPA-associated CDI in our cohort, 42.9% had positive myeloperoxidase (MPO-ANCA), 35.7% had positive proteinase 3 (PR3-ANCA), and 21.4% had negative serologies (**Table S4**). The BVAS score was higher in patients with positive PR3-ANCA who received more aggressive treatment, including intravenous methylprednisolone pulse therapy (80%, P=0.005), CTX (80%, P=0.719), and RTX (60%, P=0.024). However, the outcome was not optimistic for patients with positive PR3-ANCA; these patients had a higher recurrence rate than patients with positive MPO-ANCA or negative serologies (80% vs. 0% vs. 33.3%, P=0.022).

We studied 71 patients from former case reports, case series and retrospective studies (2–5, 12–38) (**Table 3**), 29 of whom were Asian and 42 Western. By integrating our cohort and published data on PUMCH (six patients had been included in previous studies) (3, 39), we included 20 patients from 2000 to 2022. Asian patients were older (48 ± 16 vs. 40 ± 13 years) and had a higher MPO-ANCA positivity (31% vs. 0%) than Western patients. There was no significant difference between the two groups in terms of systemic involvement. Glucocorticoids were administered to all Asian patients (100%) and most Western patients (95.2%). CTX administration was higher in Asian countries (89.7% vs. 61.9%); however, the difference was not statistically significant. Over 50% of the patients showed improvement after treatment, approximately one-sixth relapsed, and one-tenth died. Approximately half of the patients had persistent CDI, one-fifth improved with the decreased need for desmopressin replacement therapy, and one-tenth persistently required full doses.

**Table 3:** Comparison of GPA-associated DI among Asian (include this study) and western countries.

Variables	Asian countries (n=29)	Western countries (n=42)
Sex (Male)	13(44.8%)	10(23.8%)
Age	48 ± 16	40 ± 13
ANCA		
PR3	9(31.0%)	25(59.5%)
MPO	10(31.0%)	0(0%)
System Involvement		
ENT	25(86.2%)	36(85.7%)
Eye	14(48.3%)	13(31%)
Kidney	6(20.7%)	19(45.2%)
Lung	20(69.0%)	19(45.2%)
Skin	4(13.8%)	6(14.3%)
Abnormal MRI	26(89.7%)	35(83.3%)
Anterior pituitary hypofunction	12(41.4%)	22(52.4%)
Treatment		
Glucocorticoids	29(100%)	40(95.2%)
Cyclophosphamide	26(89.7%)	26(61.9%)
Rituximab	4(13.8%)	5(11.9%)
Outcome		
Improved	18(62.0%)	31(73.8%)
Recurrence	6(20.7%)	5(11.9%)
Died	4(13.8%)	2(4.8%)
Diabetes insipidus		
Improved	8(27.6%)	6(14.3%)
Resolved	4(13.8%)	6(14.3%)
Persistent	11(37.9%)	20(47.6%)

GPA, granulomatosis with polyangiitis; ANCA, anti-neutrophil cytoplasmic antibodies; MPO, myeloperoxidase; PR3, proteinase 3; ENT, Ear, Nose and Throat; MRI, Magnetic resonance imaging.

### Difference between AAV with and without CDI

The critical difference between AAV patients with and without CDI matched by sex and age by Model 1 was AAV classification (**Table S5**). GPA was more common in patients with AAV-associated CDI (87.6% vs. 33.8%), whereas MPA was more common in patients with AAV without CDI (6.3% vs. 38.8%, P=0.007). After adjustment for sex, age, and AAV classification by Model 2, patients with AAV-associated CDI had more ENT and eye involvement (81.3% vs. 35%, P<0.001; 62.5% vs. 11.3%, P<0.001, respectively) and less renal involvement (18.8% vs. 63.3%, P<0.001) than those in the non-CDI group. The CDI group had a lower U-RBC value (2.8 vs. 13.5 Cells/μL, P=0.014) and a higher eGFR (121.8 vs. 73.7 ml/min/1.73 m2, P=0.029) than the non-CDI group. There were no statistically significant differences in treatment or prognosis between the two groups.

### Risk factors for death and recurrence

**Figure 3:**
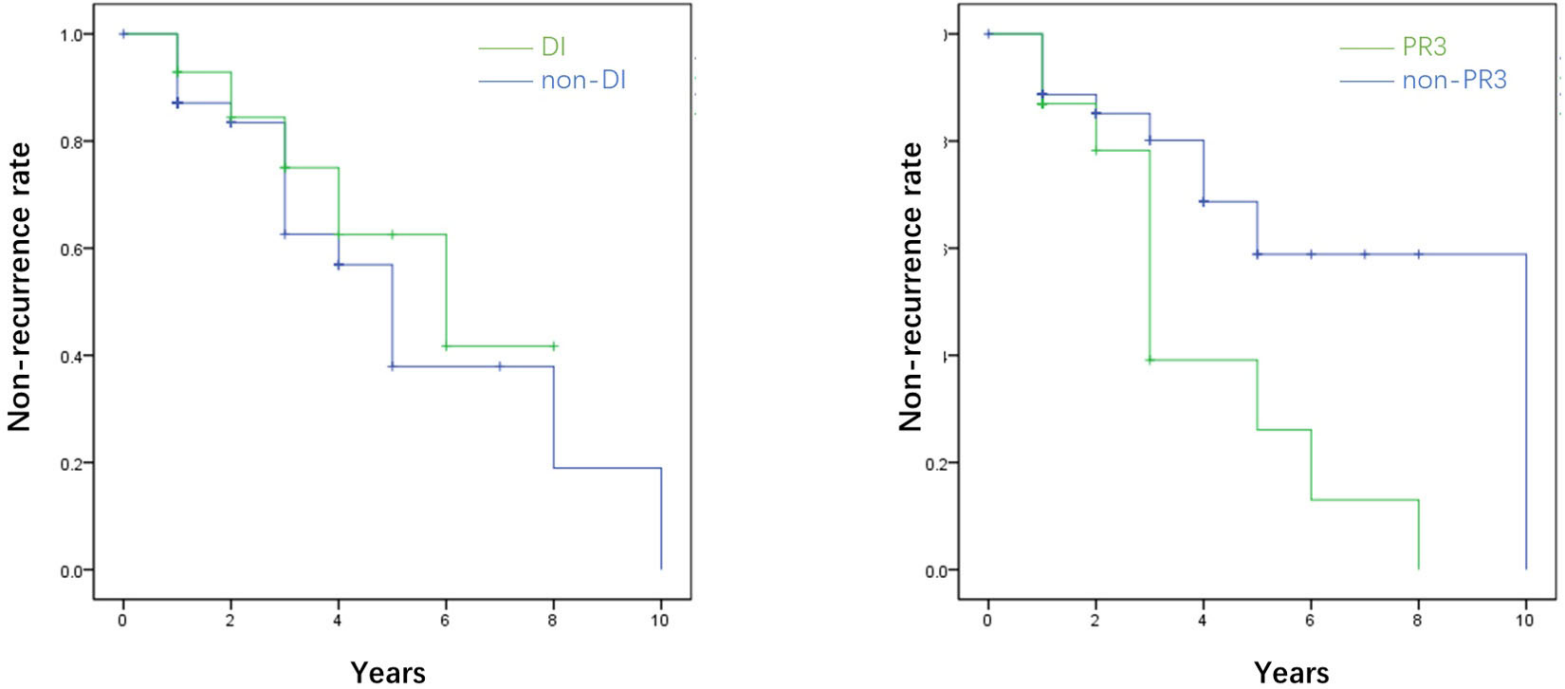
The survival analysis of the recurrence rate of AAV in terms of diabetes insipidus (DI) and proteinase 3 (PR3). The survival analysis showed that diabetes insipidus was not associated with the recurrence of AAV (P=0.471), while PR3 was the promising risk factor for predicting the recurrence of AAV (P=0.025). *AAV, ANCA-associated.

No risk factors between the death and survival groups were identified in patients with AAV-associated CDI. Among six patients who suffered from AAV recurrence, and half of them had the recurrence of CDI. Kaplan–Meier curves suggested that CDI was not associated with AAV recurrence (P=0.471), whereas PR3-ANCA positivity was a risk factor for effectively predicting recurrence in patients with AAV-associated CDI (P=0.025) (**Figure 3**).

## Discussion

We reported 16 cases of AAV-associated CDI and reviewed 71 previously reported cases. Over half of the patients had complete or partial remission with steroid treatment augmented with cyclophosphamide or rituximab. MPO-ANCA positivity was more common in Asian countries, whereas PR3-ANCA positivity prevailed in Western countries. PR3-ANCA positivity indicated a higher disease activity with a higher BVAS score, resulting in a poorer prognosis.

In our cohort, GPA accounted for 87.5% of patients with AAV-associated CDI. The incidence of pituitary involvement with GPA was 3.9% (3). There were a few cases of pituitary involvement with MPA or EGPA (12.5%). Hypertrophic pachymeningitis and CDI were associated with PR3-ANCA or MPO-ANCA positivity (7, 40); however, the detailed mechanisms of these relationships remain unclear.

CDI was present in 4% of the 357 patients with GPA in our cohort. This was higher than in the cohort of Mayo Clinic (5) (1.3%, **Table S6**), which were similar in terms of percentages for sex and age, therapeutic strategies, remission rates (87.5% vs. 50%, P=0.079); however, PR3-ANCA-positivity was relatively lesser (87.5% vs. 35.7%, P=0.019). Additionally, the complete resolution rate of CDI (meaning no longer requiring replacement therapy) was significantly higher in the Mayo Clinic (66.6% vs. 10%, P=0.018). The difference of remission rates between Mayo Clinic and PUMCH may be attributed to racial differences in foreign and Asian population; however, it is difficult to explain the low remission rate in Western patients (14.7%) at 10.5 months of follow-up (21). The systemic disease was alleviated in AAV patients and showed improvement on pituitary imaging; however, recovery of pituitary function was rare (3, 39), which was consistent with our cohort.

To investigate racial differences between Asian and Western countries, we summarized a literature review from previous studies on GPA-associated CDI (2–5, 12–38) (**Table 3**). Asian countries included China, Japan and India, and Western countries included America, Britain, the Netherlands, Canada, Turkey, France, Australia and Italy. MPO-ANCA positivity was more common in Asian countries, whereas PR3-ANCA positivity prevailed in Western countries. Even in AAV patients without CDI, MPO-ANCA is more common in southern regions of Europe and Asia, whereas PR3-ANCA is more common in northern parts of the world (41). Our data indicated that patients with GPA-associated CDI with PR3-ANCA positivity had higher BVAS scores and predicted risk of relapse even after treatment, which was also supported by the RAVE trial (42). Genome-wide association studies (GWAS) in European countries indicated that the different genetic backgrounds between patients with PR3-ANCA-positive and MPO-ANCA-positive vasculitis, which were dominant in HLA-DP and HLA-DQ variants, respectively (43). Further genomic studies on the Asian population may help elucidate the population-specific differences between Asian and Western countries.

This study has a few limitations. First, this was a single-center retrospective study which limits the external validity of the findings. Additionally, the population included in our study is hospitalized patients rather than outpatients with relatively more severe clinical condition. Moreover, AAV-associated CDI is a rare condition; therefore, prolonged follow-up periods and larger cohorts are required to illustrate the role of PR3-ANCA positivity as a risk factor for predicting recurrence.

## Conclusion

Our findings from the nested case-control study and comparison between the Asian and Western populations revealed that patients with GPA-associated CDI in Asia tend to be older, have more ENT involvement, and have less renal impairment. MPO-ANCA positivity was more in Asian patients than in patients in Western countries, whereas PR3-ANCA positivity predicted a higher recurrence rate among these patients. Further genomic studies are required to illustrate the population differences between Asian and Western countries.

## Data availability statement

The original contributions presented in the study are included in the article/**Supplementary Material**. Further inquiries can be directed to the corresponding authors.

## Ethics statement

The studies involving human participants were reviewed and approved by Peking Union Medical College Hospital (No. JS 3527). The patients/participants provided their written informed consent to participate in this study. Written informed consent was obtained from the individual(s) for the publication of any potentially identifiable images or data included in this article.

## Author contributions

XC and SZ were responsible for the conception and design of the study. XC drafted the manuscript. XP, XS, and HW were responsible for data acquisition and analysis. YW, XT and YQ provided the patients and participated in manuscript revision. HZ and LC read and approved the final version of the manuscript. All authors contributed to the article and approved the submitted version.
