# Supplementary material for: Clinical characteristics and primary outcomes of patients with ANCA-associated vasculitis and central diabetes insipidus
Source: Front Endocrinol (Lausanne). 2023 May 12;14:1173903. doi: 10.3389/fendo.2023.1173903 (PMC10213315; doi:10.3389/fendo.2023.1173903)
Supplement: Supplementary file 1 [file Table_1.docx]

sTable 1. Comparison of pituitary MRI in AAV patients with diabetes insipidus before and after treatment.

|  | Before treatment | | | | | | After treatment | | | | |
| --- | --- | --- | --- | --- | --- | --- | --- | --- | --- | --- | --- |
| ID | Pituitary size^*^ (mm) | Occupying lesion (mm) | Pituitary stalk size^#^ (mm) | Posterior hyperintense signal | PST | HCP | Follow-up time (months) | Pituitary size^*^ (mm) | Occupying lesion (mm) | Pituitary stalk size^#^ (mm) | Pituitary lesion shrink |
| 1 | 16.2×6.2×9.8 |  | 1.8×1.5 | Absence | No | Yes | NA |  |  |  | NA |
| 2 | 9.6×9.7×10.7 |  | 2.3×1.8 | Absence | No | No | NA |  |  |  | NA |
| 3 | 17.6×3.0×11 | 11.8×8.0×7.9 | 2.3×1.6 | Absence | No | No | 2 | 12.3×4.3×10.7 | no lesions | 1.4×2 | Yes |
| 4 | 13.4×7.6×12.4 |  | 2.7×2.5 | Absence | No | No | NA |  |  |  | NA |
| 5 | Normal imaging |  |  | Presence | No | No |  |  |  |  | NA |
| 6 | NA | 16×11×8 |  | Absence | Yes | Yes | Surgery |  |  |  | 1 |
| 7 | 17.2×4.9×7.3 |  | 3.9×2.3 | Absence | Yes | No | 12 | 14.8×6×7.7 |  | 3.9×1.6 | Yes |
| 8 | Normal imaging |  |  | Absence | No | No | NA |  |  |  | NA |
| 9 | 13×8.8×12.4 | 5.7×7.8 | 5.3×5.7 | Absence | Yes | No | 2 | 10.5×4.3×11.2 | 3.8×4.5 | 2.3 | Yes |
| 10 | Pituitary necrosis with local hemorrhage | | | Absence | No | No |  |  |  |  | NA |
| 11 | 15.2×11.2×10 |  | 2×2 | Absence | No | No | 2 |  |  |  | Yes |
| 12 | Normal imaging |  |  | Absence | No | No |  |  |  |  | NA |
| 13 | Normal imaging |  |  | Absence | No | Yes |  |  |  |  | NA |
| 14 | 19.9×9.3×11.3 | 7.0×4.5 | 1.8×2.3 | Absence | No | Yes | 24(relapse) | 13×7×7.5 |  | 2.2×2.3 | Yes |
| 15 | 19.2×5.1×16.2 | 10.7×7.8 | 2.3×1.8 | Absence | No | No | NA |  |  |  | NA |
| 16 | 15.1×9.7×13.5 |  | 2.8×2.6 | Absence | No | Yes | 6 | 12.6×5×11 |  | 2.8×1.9 | Yes |

^*^Pituitary size (transverse diameter, height, anteroposterior diameter); ^#^Pituitary stalk size (transverse diameter, anteroposterior diameter)

AAV, ANCA-associated vasculitis; PST, Pituitary stalk thickening; HCP, Hypertrophic cranial pachymeningitis; NA, Not Available.

sTable 2. The endocrine dysfunction in AAV patients with diabetes insipidus.

| ID | hypopituitarism | HH | secondary hypothyroidism | Hypocortisolism | GHD | hyperprolactinemia |
| --- | --- | --- | --- | --- | --- | --- |
| 1 | Yes | No | Yes | NA | No | No |
| 2 | No | No | No | No | No | No |
| 3 | No | No | No | No | No | Yes |
| 4 | No | No | No | No | NA | NA |
| 5 | No | No | No | No | No | Yes |
| 6 | Yes | Yes | Yes | Yes | NA | No |
| 7 | Yes | Yes | No | NA | NA | NA |
| 8 | Yes | No | Yes | NA | NA | NA |
| 9 | Yes | No | Yes | NA | No | NA |
| 10 | Yes | Yes | Yes | NA | Yes | No |
| 11 | Yes | No | Yes | Yes | No | Yes |
| 12 | No | No | No | NA | No | NA |
| 13 | No | No | No | NA | NA | NA |
| 14 | Yes | No | Yes | NA | No | Yes |
| 15 | No | No | No | NA | No | NA |
| 16 | Yes | Yes | Yes | No | No | No |

AAV, ANCA-associated vasculitis; HH, Hypogonadotropic hypogonadism; GHD, Growth hormone deficiency; NA, Not Available.

sTable 3. The diagnosis of AAV with histopathological examinations.

| ID | Biopsy | Detail |
| --- | --- | --- |
| 1 | 1 | Excision of intravertebral lesions, Local tissue necrosis with granuloma formation |
| 2 | 0 | NA |
| 3 | 0 | NA |
| 4 | 0 | NA |
| 5 | 1 | Sinus Biopsy, Chronic inflammation of the sinuses with fibrous tissue hyperplasia |
| 6 | 1 | Pituitary Biopsy, Granulomatous inflammation of the pituitary gland |
| 7 | 0 | NA |
| 8 | 1 | Renal biopsy, Tubulointerstitial renal damage, benign small renal artery sclerosis |
| 9 | 1 | Pulmonary Biopsy, Multiple nodular cavities with granulomas and necrosis on pathology |
| 10 | 0 | NA |
| 11 | 0 | NA |
| 12 | 0 | NA |
| 13 | 0 | NA |
| 14 | 0 | NA |
| 15 | 0 | NA |
| 16 | 1 | Bronchoscopy, eosinophilic infiltration, scattered multinucleated giant cells, may granuloma |

NA, not available.

sTable 4. The characteristics and outcome of GPA patients with diabetes insipidus among different ANCA.

|  | MPO（n=6） | PR3（n=5） | Negative（n=3） | *P* value |
| --- | --- | --- | --- | --- |
| Sex（Male） | 2（33.3%） | 4（80%） | 2（66.7%） | 0.277 |
| Presented as DI | 4（66.7%） | 2（40%） | 1（33.3%） | 0.549 |
| Presented as AAV | 2（33.3%） | 4（80%） | 3（100%） | 0.095 |
| Infection | 4（66.7%） | 5（100%） | 1（33.3%） | 0.122 |
| Thrombosis | 2（33.3%） | 1（20%） | 2（66.7%） | 0.406 |
| System involvement |  |  |  |  |
| ENT | 5（83.3%） | 4（80%） | 2（66.7%） | 0.844 |
| Eye | 3（50%） | 2（40%） | 3（100%） | 0.226 |
| Lung | 2（33.3%） | 4（80%） | 0 | 0.071 |
| Skin | 4（66.7%） | 2（40%） | 0 | 0.24 |
| Neuropathy | 2（33.3%） | 1（20%） | 3（100%） | 0.071 |
| Kidney | 0 | 2（40%） | 0 | 0.122 |
| Cardiovascular | 0 | 1（20%） | 0 | 0.379 |
| Treatment |  |  |  |  |
| Corticosteroids | 7（100%） | 5（100%） | 3（100%） | 1 |
| MP pulse. | 0 | 4（80%） | 3（100%） | 0.005 |
| CTX | 5（83.3%） | 4（80%） | 3（100%） | 0.719 |
| RTX | 0 | 3（60%） | 0 | 0.024 |
| MTX | 2（33.3%） | 1（20%） | 0 | 0.514 |
| MMF | 1（16.7%） | 1（20%） | 0 | 0.379 |
| FK506 | 0 | 1（20%） | 0 | 0.719 |
| IVIG | 1（16.7%） | 0 | 0 | 0.488 |
| BVAS | 14(12-16) | 27(17-37) | 16.5(16-17) | 0.344 |
| Outcome |  |  |  |  |
| Improved | 5（83.3%） | 1（20%） | 1（33.3%） | 0.091 |
| Recurrence | 0 | 4（80%） | 1（33.3%） | 0.022 |
| Dead | 1（14.3%） | 0 | 1（33.3%） | 0.417 |

*AAV, ANCA-associated vasculitis; GPA, granulomatosis with polyangiitis; DI, diabetes insipidus; ANCA, anti-neutrophil cytoplasmic antibodies; MPO, myeloperoxidase; PR3, proteinase 3; ENT, Ear, Nose and Throat; MP pulse., intravenous methylprednisolone pulse therapy; PDN po., oral prednisone; RTX, rituximab; CTX, cyclophosphamide; MTX, methotrexate; FK506, tacrolimus/rapamycin; MMF, mycophenolate mofetil; IVIG, intravenous immune globulin.

sTable 5. The difference between AAV with and without diabetes insipidus.

|  |  | Model 1 | | Model 2 | |
| --- | --- | --- | --- | --- | --- |
|  |  | (Matched by sex and age) | | (Matched by sex, age and AAV classification) | |
|  | DI（n=16） | non-DI（n=80） | *P* value | non-DI（n=80） | *P* value |
| System Involvement | |  |  |  |  |
| ENT | 13（81.3%） | 18（22.5%） | <0.001 | 28（35.0%） | <0.001 |
| Eye | 10（62.5%） | 5（6.3%） | <0.001 | 9（11.3%） | <0.001 |
| Lung | 7（43.8%） | 42（52.5%） | 0.52 | 49（61.3%） | 0.191 |
| Kidney | 3（18.8%） | 52（65%） | <0.001 | 49（61.3%） | <0.001 |
| AAV classification | |  | 0.007 |  | 1 |
| GPA | 14（87.6%） | 27（33.8%） |  | 65（81.3%） |  |
| MPA | 1（6.3%） | 31（38.8%） |  | 5（6.3%） |  |
| EGPA | 1（6.3%） | 17（21.3%） |  | 5（6.3%） |  |
| ANCA |  |  |  |  |  |
| p-ANCA | 4（25%） | 30（37.5%） | 0.34 | 17（21.3%） | 0.74 |
| c-ANCA | 6（37.5%） | 14（17.5%） | 0.072 | 28（35.0%） | 0.849 |
| MPO | 5（31.3%） | 32（40%） | 0.512 | 17（21.3%） | 0.385 |
| PR3 | 4（25%） | 14（17.5%） | 0.483 | 34（42.5%） | 0.191 |
| BUN（mmol/L） | 4.4(3.1-5.0) | 7.9(5.1-16.4) | 0.003 | 5.9(4.2-9.2) | 0.012 |
| Scr（μmol/L） | 58.0(48.0-64.3) | 97.0(68.0-207.0) | 0.016 | 98.5(57.5-72.8) | 0.011 |
| eGFR（ml/min/1.73m2） | 121.8(101.6-136.8) | 73.5(29.5-102.1) | 0.004 | 73.7(28.6-114.7) | 0.025 |
| UA（μmol/L） | 263.5(213.3-362.8) | 281.0(213.0-452.0) | 0.708 | 251.5(199.8-348.3) | 0.88 |
| Alb（g/L） | 37.8±1.0 | 34.0±0.7 | 0.016 | 34.2±0.7 | 0.029 |
| TCO2（mmol/L） | 29.1(27.8-30.3) | 26.8(25.0-28.7) | 0.001 | 25.9(24.9-28.5) | 0.003 |
| 24hUP（g/24h） | 0.1(0.1-0.3) | 0.5(0.1-1.6) | 0.016 | 0.5(0.1-1.0) | 0.021 |
| U-RBC（Cells/μL） | 2.8(1.1-8.9) | 8.4(3.0-84.9) | 0.011 | 13.5(2.9-132.3) | 0.014 |
| C3（g/L） | 1.3±0.1 | 1.1±0.1 | 0.059 | 1.1±0.1 | 0.082 |
| C4（g/L） | 0.2±0.1 | 0.2±0.1 | 0.245 | 0.2±0.1 | 0.485 |
| D-Dimer（mg/L） | 0.9±0.3 | 2.7±0.5 | 0.006 | 3.1±0.6 | 0.021 |
| ESR（mm/h） | 34.9±7.4 | 45.2±4.3 | 0.275 | 49.0±4.1 | 0.131 |
| CRP（mg/L） | 31.4±10.1 | 30.1±5.3 | 0.922 | 37.6±6.2 | 0.673 |
| Comorbidities | |  |  |  |  |
| Thrombosis | 3（17.8%） | 6（7.5%） | 0.37 | 5（6.3%） | 0.22 |
| Infection | 6（37.5%） | 18（22.5%） | 0.206 | 25（31.3%） | 0.625 |
| Anemia | 5（31.3%） | 17（21.3%） | 0.137 | 22（27.6%） | 0.155 |
| Treatment |  |  |  |  |  |
| PDN po. | 15（93.8%） | 78（97.5%） | 0.431 | 76（95.0%） | 0.837 |
| MP pulse. | 4（25%） | 26（32.5%） | 0.555 | 21（26.3%） | 0.917 |
| CTX | 11（68.8%） | 63（78.8%） | 0.385 | 58（72.5%） | 0.761 |
| RTX | 1（6.3%） | 5（6.3%） | 0.648 | 7（8.8%） | 0.741 |
| MMF | 1（6.3%） | 3（9.4%） | 1 | 5（6.3%） | 1 |
| PE | 1（6.3%） | 1（1.3%） | 0.21 | 0 | 0.025 |
| Dialysis | 1（6.3%） | 6（7.5%） | 0.861 | 6（7.5%） | 0.861 |
| Length of stay(d) | 30(20-38) | 24(16-33) | 0.155 | 23(16-30) | 0.279 |
| BVAS | 16.5±1.8 | 15.6±1.0 | 0.615 | 13.8±1.5 | 0.243 |
| Outcome |  |  | 0.513 |  | 0.52 |
| Improved | 8（50%） | 49（61.3%） |  | 47（58.8%） |  |
| Recurrence | 5（31.3%） | 28（35.0%） |  | 31（38.7%） |  |
| Death | 1（6.3%） | 1（1.3%） |  | 2（2.5%） |  |

*AAV, ANCA-associated vasculitis; GPA, granulomatosis with polyangiitis; MPA, microscopic polyangiitis; EGPA, eosinophilic granulomatosis with polyangiitis; DI, diabetes insipidus; ANCA, anti-neutrophil cytoplasmic antibodies; MPO, myeloperoxidase; PR3, proteinase 3; ENT, Ear, Nose and Throat; WBC, white blood cells; L, Lymphocyte; Hb, hemoglobin; PLT, blood platelet; BUN, blood urea nitrogen; Scr, serum creatinine; UA, uric acid; TCO2, carbon dioxide binding capacity; Alb, albumin; 24h UP, 24h urine protein; U-RBC, urine red blood cells;C3, complement component 3; C4, complement component 4; ESR, erythrocyte sedimentation rate; CRP, C-Reactive Protein; MP pulse., intravenous methylprednisolone pulse therapy; PDN po., oral prednisone; RTX, rituximab; CTX, cyclophosphamide; FK506, tacrolimus/rapamycin; MMF, mycophenolate mofetil; PE, plasma exchange; BVAS, Birmingham Vasculitis Activity Score (BVAS) v3.

sTable 6. Comparison between the research published by Mayo Clinic and this study.

| Institution | Mayo Clinic | Peking Union Medical College Hospital |
| --- | --- | --- |
| Year | 1996-2011 | 2012-2022 |
| Patients | 637 GPA | 357 GPA |
| Pituitary disease | 8 patients | 14 patients |
| Diabetes insipidus | 6 patients (1.3%), other 2 possible DI | 14 patients (4.0%) |
| Hypopituitarism | 7/8 (87.5%) | 9/16 (56.3%) |
| Following time | 76(range 18-132) months | 48(range 12-120) months |
| Sex (Male) | 4/8 (50%) | 8/14 (57.1%) |
| Age | 48(28,68) | 49(36,60) |
| ANCA | 7/8 PR3 | 6/14 MPO, 5/14 PR3, 3/14 Negative |
| Eye, nose and throat | 8/8(100%) | 11/14(78.6%) |
| Kidney | 4/8(50%) | 2/14(14.3%) |
| Lung | 4/8(50%) | 6/14(42.9%) |
| Eye | NA | 8/14(57.1%) |
| Skin | 2/8(25%) | 5/14(35.7%) |
| Cardiovascular | 1/8(12.5%) | 1/14(7.1%) |
| Glucocorticoids | 8/8(100%)) | 14/14(100%) |
| Cyclophosphamide | 6/8(75%); 4/6 remission | 12/14(85.7%); 5/12 remission |
| Rituximab | 3/8(37.5%); 3/3 remission | 3/14(21.4%); 3/3 remission |
| Outcome | 7/8(87.5%) remission | 2/14 died, 7/14 remission, 5/14 relapse |
| DI resolved | 4/6(66.7%) | 1/10(10%) |
| DI improved | 1/6(16.7%) | 6/10(60%) |
| DI persistent | 1/6(16.7%) | 3/10(30%) |
| Secondary hypogonadism improved | 4/7(57.1%) | 2/9(22.2%) |

DI, diabetes insipidus; ANCA, anti-neutrophil cytoplasmic antibodies.
